# Outpatient Interventions That May Enhance the Care of a Patient with Co-existing Moyamoya and Down Syndromes

**DOI:** 10.7759/cureus.2336

**Published:** 2018-03-16

**Authors:** Anita Lwanga, Waldo Herrera, Katya Cruz Madrid, Antony Irungu

**Affiliations:** 1 Department of Academic Internal Medicine and Geriatrics, University of Illinois at Chicago; 2 Internal Medicine/division of Hoapital Medicine, NorthShore University Health System; 3 Department of Academic Internal Medicine and Geriatrics, University of Illinois In Chicago; 4 Medical Student, Ross University School of Medicine

**Keywords:** moyamoya syndrome, down syndrome, progeroid syndrome, geriatric syndrome

## Abstract

Moyamoya vasculopathy is a condition of chronic, progressive occlusion of the distal internal carotid arteries and the Circle of Willis. The resultant ischemia produces compensatory angiogenesis and the growth of a network of collateral blood vessels, which on angiography resemble a “puff of smoke” or “moyamoya” in Japanese. The objective of this case report is to describe the clinical course of a patient with Down and moyamoya syndromes and to enlighten clinicians about strategies that can be taken to enhance the care of similar patients.

A 55-year-old African American female presented to the hospital with complaints of headache, vision loss, dysarthria, and ataxia. She had a past medical history of Down syndrome and a stroke with residual lower extremity weakness. At her baseline, the patient was able to perform her activities of daily living but required assistance with independent activities of daily living. Computed tomography of the brain showed hypodense areas at the right occipital, temporal, and parietal lobes. Computed tomography angiography of the head and neck identified occlusion bilaterally at the supraclinoid internal carotid arteries and right posterior cerebral artery; there was collateral arterial flow within the right middle cerebral and anterior cerebral arteries that was consistent with moyamoya vasculopathy.

Patients with Down syndrome experience premature accelerated aging and suffer from comorbidities seen in geriatric patients by the time they reach their 40s. Patients with moyamoya vasculopathy experience neurocognitive and neuropsychiatric deficits that correspond to the regions of the brain that are affected. This patient with Down and moyamoya syndromes had impaired neurocognitive and functional status, and we believe that she would have benefited from receiving a comprehensive geriatric assessment and neuropsychiatric testing.

## Introduction

Moyamoya disease is a rare condition of progressive stenosis and occlusion of the internal carotid arteries and the Circle of Willis [1-3]. The resultant ischemia and hypoxia produce compensatory angiogenesis that stimulates the growth of a network of tiny, branched collateral blood vessels [1-2]. On angiography, this network of blood vessels resembles a “puff of smoke” or “moyamoya” in the Japanese language, which is where the condition derives its name [1].

In the United States (US), the incidence of moyamoya is approximately 0.086/100,000 individuals [4]. It is more common in females, individuals of Asian descent, and has a bimodal age distribution with peaks in the first and fourth decades of life [3-4]. Moyamoya vasculopathy is uncommon in African Americans; those of African descent that are affected are usually younger and have co-existing sickle cell disease [4]. In the US, concomitant moyamoya and Down syndromes occur more commonly in individuals of European and South American ancestry; the combination of moyamoya vasculopathy and Down syndrome is rarely seen in those of African descent [5].

Moyamoya can be congenital, acquired, or idiopathic [1]. The pathophysiology is not well understood, and the cause remains unknown [2]. In cases where the individual has a preexisting hemoglobinopathy, autoimmune disorder, meningeal infection, congenital syndrome, or vascular disease associated with moyamoya, the diagnosis is moyamoya syndrome rather than moyamoya disease [1-2].

Moyamoya is often initially identified on computed tomography (CT) or magnetic resonance angiography (MRA) of the brain after patient a presents with complaints of headaches, cerebral vascular event (CVA), transient ischemic attack, or seizure [1-2, 6]. In unilateral cases, catheter angiography is required to confirm the diagnosis [7]. 

In general, urgent revascularization is the first line in managing patients with moyamoya; if revascularization is performed rapidly, it can improve outcomes [2]. Management of patients with co-existing Down and moyamoya syndromes can be more complex [6]. At their baseline, patients with Down syndrome have functional impairments which, when added to neurological deficits from moyamoya, make it difficult to complete a comprehensive neurological assessment; furthermore, patients with Down syndrome are predisposed to atlantoaxial instability and congenital cardiac anomalies that increase the risk of adverse outcomes in the perioperative period [6]. With these issues in mind, Nussbaum et al. recommended cerebral revascularization in selected cases [6]. Regardless of the intervention, the patient’s neurological status at the time of treatment is the most important prognostic factor [2].

The objective of this case report is to describe the clinical course of a patient with Down and moyamoya syndromes and to enlighten clinicians about strategies that can be implemented to improve the medical care of similar patients.

## Case presentation

A 55-year-old African American female was brought to the emergency department for evaluation of headaches, vision loss, dysarthria, and ataxia. She had a past medical history of Down syndrome and a CVA with mild residual lower extremity weakness. She lived with her sister and was able to perform her activities of daily living on her own but needed assistance with her independent activities of daily living. Her family history was unremarkable. She did not have allergies. Her preadmission medications included aspirin and atorvastatin.

On initial evaluation, the patient’s blood pressure was 144/85; the rest of her vital signs were within normal limits. She was obese and had Down-like facies. Her neurological exam was remarkable for left-sided facial droop, right gaze preference, vision loss, severe dysarthria, 3/5 strength at the left upper extremity, and 2/5 strength at the left lower extremity; we were unable to complete the rest of the neurological exam because of poor cooperation. Her cardiovascular, respiratory, abdominal, and skin exams were unremarkable.

**Figure 1:**
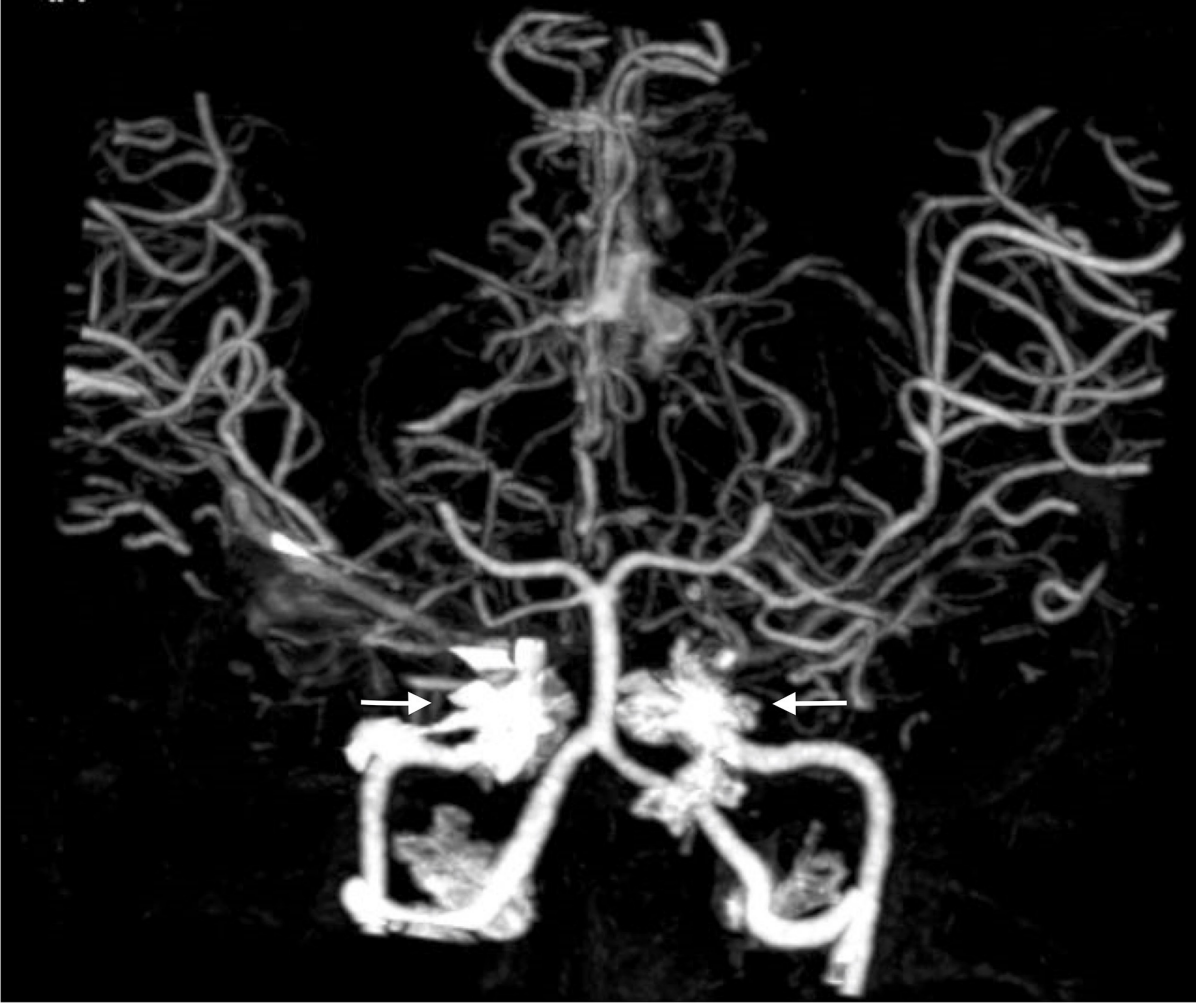
Computerized tomographic angiography (CTA) of the head and neck with contrast demonstrating occlusion of the bilateral supraclinoid internal carotid arteries

**Figure 2:**
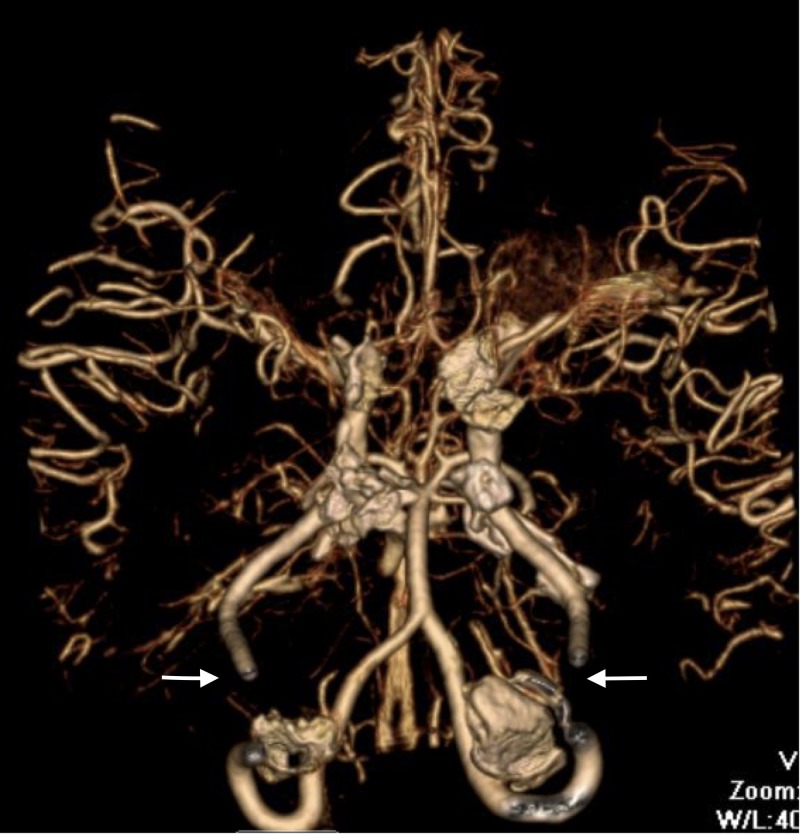
Additional image from the computerized tomographic angiography (CTA) of the head and neck illustrating bilateral occlusion of the internal carotid arteries

A CT scan of the brain revealed hypodense areas at the right occipital, temporal, and parietal lobes. Computed tomography angiography (CTA) of the head and the neck identified occlusions bilaterally at the supraclinoid internal carotid arteries and right posterior cerebral artery; there was collateral arterial flow within the right middle cerebral and anterior cerebral arteries that was consistent with moyamoya (Figures 1-2). The patient was transferred to a tertiary facility for revascularization. We were unable to follow her course once she was transferred.

## Discussion

Progeroid syndromes are a group of genetic conditions associated with accelerated and premature aging [8]. Down syndrome, one of the segmental progeroid syndromes, affects the central nervous system, vision, hearing, immune system, endocrine system, respiratory system, gastrointestinal tract, urinary tract, and musculoskeletal system [8]. By the time they are in their forties, many individuals with Down syndrome have graying hair, hair loss, variations in adipose tissue distribution, amyloidosis, cataracts, autoimmune conditions, neuropathological changes seen in Alzheimer’s disease, osteoporosis, geriatric syndromes, functional deficits, and social problems that commonly occur in older adults [8-10]. The mechanism underlying premature aging in Down syndrome is currently unknown [8].

The majority of patients with moyamoya vasculopathy experience neurocognitive and neuropsychiatric deficits that correspond the regions of their brain that have been affected [1]. The cognitive impairments are broad and may affect intelligence, processing speed, visual-spatial performance, and executive function; mild depression may also occur [1]. Patients affected by moyamoya should receive comprehensive neuropsychiatric testing to fully assess their cognitive deficits [1].

As a result of Down syndrome and a CVA, the patient described in this case report had neurocognitive and functional deficits that often occur in geriatric patients. Moyamoya vaso-occlusive disease further compounded her deficits and predisposed her to develop additional geriatric syndromes. In order to address her socioeconomic, nutritional, neurological, and psychiatric issues, as well as prepare her family for the challenges associated with premature aging, we believe that the patient would have benefited from a comprehensive geriatric assessment and neuropsychiatric testing in an outpatient setting [1, 8]. Unfortunately, we were unable to follow her course after she was transferred.

## Conclusions

Patients with Down syndrome experience accelerated aging. Patients with moyamoya syndrome experience neurocognitive and neuropsychiatric deficits that correspond the regions of the brain that are affected. After receiving treatment for moyamoya vasculopathy, individuals with co-existing Down and moyamoya syndromes should have a comprehensive geriatric assessment and neuropsychiatric testing to address the unique challenges that they face.
